# A comparison of MITS counseling and informed consent processes in Pakistan, India, Bangladesh, Kenya, and Ethiopia

**DOI:** 10.1186/s12978-020-00969-w

**Published:** 2020-08-12

**Authors:** Anam Shahil Feroz, Christina Paganelli, Milka Bunei, Beza Eshetu, Shahana Parveen, Sayyeda Reza, Chaitali Sanji, Shiyam Sunder Tikmani, Shivaprasad S. Goudar, Guruprasad Goudar, Sarah Saleem, Elizabeth M. McClure, Robert L. Goldenberg

**Affiliations:** 1 The Aga Khan University – Department of Community Health Sciences, Stadium Road, PO Box 3500, Karachi, 74800 Pakistan; 2 RTI International, Seattle, WA USA; 3 Washington State University, GH Kenya, Nairobi, Kenya; 4 Ababa University, College of Health Science, Addis Ababa, Ethiopia; 5 icddr,b, Dhaka, Bangladesh; 6 Jagadguru Jayadeva Murugharajendra Medical College, Davangere, Karnataka India; 7 KLE Academy of Higher Education and Research’s J N Medical College, Belagavi, Karnataka India; 8 Bapuji Education Association’s JJM Medical College, Davangere, Karnataka India; 9 RTI International, Research Triangle Park, NC USA; 10 Columbia University – Department of Obstetrics and Gynecology, New York, USA

**Keywords:** MITS counseling, Informed consent processes, Comparative, PURPOSe Pakistan, PURPOSe India, CHAMPS Bangladesh, SIP Ethiopia, PRESS 2 Kenya

## Abstract

Globally, more than 5 million stillbirths and neonatal deaths occur annually. For many, the cause of death (CoD) is unknown. Minimally invasive tissue sampling (MITS) has been increasingly used in postmortem examinations for ascertaining the CoD in stillbirths and neonates. Our study compared the counseling and consent methods used in MITS projects in five countries in Africa and south Asia. Key informant interviews were conducted with researchers to describe the characteristics and backgrounds of counselors, the environment and timing of consent and perceived facilitators and barriers encountered during the consent process. Counselors at all sites had backgrounds in social science, psychology and counseling or clinical expertise in obstetrics/gynecology or pediatrics. All counsellors received training about techniques for building rapport and offering emotional support to families; training duration and methods differed across sites. Counselling environments varied significantly; some sites allocated a separate room, others counselled families at the bedside or nursing stations. All counsellors had a central role in explaining the MITS procedure to families in their local languages. Most sites did not use visual aids during the process, relying solely on verbal descriptions. In most sites, parents were approached within one hour of death. The time needed for decision making by families varied from a few minutes to 24 h. In most sites, extended family took part in the decision making. Because many parents wanted burial as soon as possible, counsellors ensured that MITS would be conducted promptly after receiving consent. Barriers to consent included decreased comprehension of information due to the emotional and psychological impact of grief. Moreover, having more family members engaged in decision-making increased the complexity of counselling and achieving consensus to consent for the procedure. While each site adapted their approach to fit the context, consistencies and similarities across sites were observed.

## Background

Globally, there are approximately 2.7 million neonatal deaths every year; with 2.6 million annual deaths, the number of stillbirths is similar [1]. In order to reach the United Nations Sustainable Development Goals (SDG) target for stillbirth and neonatal mortality of 12 deaths per 1000 live births by 2030, accurate causes of death must be identified to effectively design and implement programs to reduce preventable mortality [2].

Since 2006, minimally invasive tissue sampling (MITS) has been increasingly used to help establish causes of in stillbirths and neonates, especially infectious causes. MITS is a needle-based postmortem examination that uses very fine needles to percutaneously collect body fluids and organ tissue samples that are then analyzed through histopathological and microbiological techniques. Unlike a complete diagnostic autopsy, MITS can be performed with limited infrastructure and by trained medical technicians such as histopathologist or mortuary assistants [3]. Compared with complete diagnostic autopsy, MITS is faster, less expensive, and markedly less invasive, and has demonstrated potential to support accurate cause of death determination in stillbirths and neonates [1, 4]. MITS may be more acceptable to parents because it is non-disfiguring, and can be performed quickly, with fewer delays for the potential burial practices. MITS also provides researchers, policy-makers and society with high quality pathology-based postmortem data to inform the design and implementation of interventions to prevent neonatal deaths and stillbirths [5].

The hypothetical acceptability of MITS has also recently been assessed across diverse geographic settings, including north India [3], Kenya [6], Mali [6], Mozambique [6], Pakistan [5], and the United Kingdom [7]. These studies have demonstrated a high theoretical acceptability of MITS across a range of participants, including parents, relatives of the deceased, healthcare providers, and religious and community leaders [3, 5–7]. However, these studies have also reported some perceived barriers in the acceptability of MITS, which include lack of knowledge about the procedure, lack of trust in health professionals, lack of rapport with patients, lack of sensitivity on the part of health professionals when discussing MITS and a sense of next of kin feeling rushed in their decision making [3, 5–7]. Many of the barriers cited in recent publications have the potential to be addressed through the MITS counselling and consent processes. A detailed understanding of the current consent and decision making processes for the MITS procedure is required to overcoming perceived barriers to acceptability of MITS.

To understand the various approaches to MITS counseling and consent in stillbirth and neonatal populations, we aim to describe practical experiences of how MITS counseling has been approached at five different sites in Asia and Africa. More specifically, we sought to look at the similarities and differences in the MITS counselling and consent processes including the selection and training of MITS counsellors, attributes of the environments where counselling and consent occurred, and the specific way MITS is described and explained. Further, the study explored the decision-making process for MITS among families and identified facilitators and barriers in the consent process across these sites.

### Projects descriptions

The MITS Surveillance Alliance is a network of researchers and institutions using MITS in postmortem examination with funding from the Bill & Melinda Gates Foundation. Comprised of representatives from 25 MITS projects across 17 different countries in Africa, Asia and South America and coordination from RTI International, the MITS Surveillance Alliance aims to improve cause of death ascertainment through the expansion of MITS globally. The MITS Alliance facilitates a global MITS community of practice and convenes technical working groups to share best practices and lessons learned. Technical working groups include cause of death, pathology as well as social and behavioral sciences.

Within the Social and Behavioral Sciences Technical Working Group, five projects in the MITS Alliance were initially selected, to understand their experiences with MITS counseling and consent (Table 1). The projects included in this study were selected because their study population was focused on stillbirths and neonates. Further, these five projects represent diverse geographical regions where MITS was conducted and had progressed sufficiently enough in their study implementation to have perspectives to share. These projects include Study of Illness in Preterms (SIP) in Ethiopia, Pediatric Respiratory Etiology Surveillance Study (PRESS2) in Kenya, Project to Understand and Research Preterms and Stillbirths (PURPOSe) in India, PURPOSe Pakistan and Child Health and Mortality Prevention Surveillance (CHAMPS) study in Bangladesh. All are participants in the MITS Surveillance Alliance, an organization of investigators studying MITS in low-and-middle-income countries, however each study was distinct and had its own Principal Investigator, study protocol and funding. The information presented here is a result of key informant interviews with researchers from five MITS Alliance member projects working with stillbirth and neonatal populations. Key informant interviews with researchers were conducted to describe the characteristics and backgrounds of counsellors, the environment and timing of consent and perceived facilitators and barriers encountered during the consent process. It should be noted that each MITS project conducted their own formative assessments and those results informed the design and implementation of their consent process, described in detail elsewhere [5, 8–10].

**Table 1:** Overview of projects

Project name	Sample population age range	Country
PURPOSe	Stillbirths and neonates	Pakistan
PURPOSe	Stillbirths- 5 years	India
CHAMPS	Stillbirths – 5 years	Bangladesh
SIP	Neonates	Ethiopia
PRESS2	1–59 months	Kenya

The Project to Understand and Research Preterm Pregnancy Outcomes and Stillbirths-in South Asia (PURPOSe) [PURPOSe Pakistan & PURPOSe India]: In South Asia, where most stillbirths and neonatal deaths occur [11], a cohort study [PURPOSe] was conducted in hospitals in Davengere, India and Karachi, Pakistan to prospectively obtain detailed information on causes of stillbirths and preterm neonatal death [10].

Child Health and Mortality Prevention Surveillance (CHAMPS) [CHAMPS Bangladesh]: A prospective study was conducted at the CHAMPS site in Baliakandi (Rajbari District), Bangladesh to identify the etiology of death among children less than five years of age. The data on cause of death is made available to policy makers and communities on a public database to inform public health programming.

Study of Illness in Preterm (SIP) [SIP Ethiopia]: A prospective, multicenter, cross-sectional, observational study was conducted in five hospitals in Addis Ababa, Gondar and Jimma, Ethiopia to define the major causes of mortality in preterm neonates [12].

Pediatric Respiratory Etiology Surveillance Study (PRESS 2) [Kenya]: A prospective study was conducted to identify causes and etiologies associated with respiratory disease-related deaths among children (age 1–59 months) with respiratory illness admitted to the Kenyatta National Hospital (KNH) in Nairobi, Kenya [13].

### Ethics approvals

Each study was reviewed by the respective ethics review committee of their institutions. All informants provided informed consent to participate in the research study.

## Main text

### Counsellors’ selection

For all projects, study staff responsible for patient consent and counselling all had backgrounds in either social sciences, psychology, counselling or clinical expertise in obstetrics, gynecology or pediatrics. In PURPOSe Pakistan, female healthcare providers including midwives, and lady health visitors, nurses with clinical backgrounds in either obstetrics, gynecology, or pediatrics and with basic skills in patient counseling were recruited for the counselling roles. In CHAMPS Bangladesh, one female and four male counsellors, aged 28–35 years, with backgrounds in social sciences or psychology were recruited and given the title ‘Field Research Officer’s Assistant’. The CHAMPS Bangladesh team were striving for more gender balance but experienced difficulties in identifying eligible female counsellors who were willing to stay at the field site (outside of the capital Dhaka). In PRESS2 Kenya, the primary study counsellor was a 40-year-old female with 17 years’ prior experience in grief counseling and a degree in psychology/counselling. The primary counsellor was responsible for several study nurses who were also involved in the counselling and consent process. In SIP Ethiopia, healthcare providers directly involved with the patient prior to death, including pediatric residents and bachelor’s prepared nurses ranging in age from 20 to 35 years old agreed to participate in the study as counsellors for the MITS procedure. In PURPOSe India, obstetrics and gynecology residents and pediatric residents, aged between 25 and 27 years, posted in the labor room and neonatal intensive care unit, respectively were selected for the counsellor roles.

### Counsellors’ training

Across all sites, counsellors participated in training for their roles as MITS counsellors. In SIP Ethiopia, the counsellors were trained on the counselling techniques and the consenting process, which helped them in approaching the parents of the deceased child. Since the counsellors were part of the health facility staff, and were directly involved in providing care to the neonates; this helped them establish good rapport with the parents and allowed easy communication. In PURPOSe Pakistan and CHAMPS Bangladesh, counsellors received one-week intensive training with on-the-job coaching and the training included didactic lectures and role-plays. The PURPOSe Pakistan team also prepared a counseling manual, which served as guidebook for training staff on counseling techniques. In PURPOSe India, the obstetrics and gynecology residents were provided with three hours training by study coordinators, once every two months. In PRESS2 Kenya, the primary study counsellor and study nurses participated in a 3-day grief counseling course.

### Counsellors’ role in the study

In all five projects, the counsellors were responsible for building rapport with the families and offering counselling support to the parents and other family members of the deceased stillbirths and neonates. In PURPOSe Pakistan and CHAMPS Bangladesh, counsellors participated in patient rounds with the attending physician as part of rapport-building strategy. However, in CHAMPS Bangladesh, frequent visits by counsellors prior to death were not well received by parents and families and therefore counsellors adjusted their procedure to wait until after a death to introduce themselves. The initial rapport was built up by study physicians and study medical technologists as they participated on ward rounds with facility physicians. Once notified of a death, the MITS physician introduced the family/guardian to the counsellor. At this time, the counsellor’s primary role was to provide grief support to the family prior to seeking informed consent. It was only after this that the counsellors proceeded with consent for MITS. In SIP Ethiopia, a counsellor first approached the parents (mother/father) and counselled them for a complete diagnostic autopsy. If they refused the complete diagnostic autopsy, the counsellor then briefed the parents and sought consent for the MITS procedure. In PURPOSe Pakistan and PRESS2 Kenya, soon after death, the counsellors approached the family about the MITS procedure and sought consent to determine the cause of death. In all settings, as soon as the death occurred, the counsellor notified study pathologists/physicians and on-duty technicians so that they could begin preparations for the pending/possible MITS procedure. The counsellors in all sites were responsible for ensuring that the body was respectfully handed over to parents following the procedure. In PRESS2 Kenya, in cooperation with the study pathologists, the study counsellor was responsible for communicating MITS results to the family. In PURPOSe India, counsellors had a very important role in explaining the study purpose, MITS procedure and its associated benefits to the parents.

### The counselling environment

In PURPOSe Pakistan, in an effort to minimize interference from other clinical activities and limit the counselling to the parents, corners in both the Emergency Room (ER) and the neonate intensive care unit (NICU) corridor were allocated for MITS counselling. While the counselling environment was not ideal, the counsellors were diligent in maintaining privacy and confidentiality of the family. In PRESS2 Kenya, a separate room at the mortuary department was allocated for counseling and ensured privacy and confidentiality for families. The room was equipped with chairs and was spacious enough to comfortably accommodate immediate family members and the counsellor. Occasionally, if the counsellor was on the wards at the time of death, counselling was conducted at the bedside or in a physician’s office in the pediatric department. In SIP Ethiopia, there were no separate rooms for the counselling. The parents were counseled either at the bedside or at the nurses’ station, near the NICU. Despite the counsellors’ attempts to safeguard parents’ privacy and confidentiality, due to the high-traffic nature of the area, the counsellors felt that at times, the parents found the counselling environment unwelcoming and overwhelming. In CHAMPS Bangladesh, initially counselling was carried out in the wards and open spaces. However, the counsellors were concerned that the setting was not sufficiently private and subsequently a new, relatively quieter place was allocated for counselling purposes. The PURPOSe India team carried out the counselling in a separate room to ensure privacy and facilitate communication between parents and the counsellors.

### Who explains the study and the consent process?

The consent process involves obtaining an informed consent from parents for conducting the MITS procedure on their deceased child. Informed consent is a process in which a health care provider educates parents about the risks, benefits, and alternatives of a procedure. The parent then makes a voluntary decision about whether to undergo the procedure. Implicit in providing informed consent is an assessment of the parent’s understanding, and documentation of the process. It is a collaborative process allowing parents and healthcare providers, and counsellors to make decisions together, accounting for the parent’s unique preferences and priorities. In SIP Ethiopia, PURPOSe Pakistan, PURPOSe India, CHAMPS Bangladesh and PRESS2 Kenya, all counsellors had a central role in explaining the study purpose and MITS procedure to families in their local languages. The counsellors explained that the procedure will help identify the cause of death and should ultimately aid the treatment of other children. While explaining the MITS procedure, the counsellors at all five sites explicitly emphasized that MITS is less-invasive [requiring only a small incision/ minimal damage of body tissue] and is carried out with a fine-gauge needle to draw small samples of tissue from body organs. Also, the parents were reassured that the intact body would be handed over to them immediately after the procedure, so that parents could proceed with burial practices. In SIP Ethiopia, if the parents desired more detailed information, the counsellors expounded on the initial description and described other details related to MITS such as how the sampled tissues would be processed for analysis. In PURPOSe Pakistan, counsellors gave parents the option to see the body immediately after MITS to assure them that the body was intact and in the same condition as was described. Few families accepted this offer and were satisfied with the counsellors’ descriptions.

### Tools used to aid counselling process

In SIP Ethiopia, PURPOSe Pakistan, PURPOSe India and PRESS2 Kenya, no visual aids were used to aid counselling and counsellors solely relied on a verbal description for families. In most cases, the CHAMPS Bangladesh team shared basic mortality data via simple mortality charts and documents containing information about grief. While not intended to provide additional insight into the MITS procedure, both the PURPOSe Pakistan and SIP Ethiopia teams had Quranic evidence in the form of written verses on permissibility of MITS procedure from the local Muslim religious leader to facilitate the counselling process. The Quranic evidence helped teams to clarify parents’ misconceptions related to permissibility of the MITS procedure in Islam. The counsellors of the CHAMPS Bangladesh obtained a Fatwa from the Islamic Foundation of Bangladesh which they shared with families at their request during the informed consent process.

### The consent and decision-making process with parents and family

- *Timing of approaching bereaved parents*

In SIP Ethiopia, CHAMPS Bangladesh, PURPOSe India and PURPOSe Pakistan, parents were approached by the counsellor within 1 h of the death. This was necessary in all four sites because families typically leave the hospital immediately following death to initiate burial practices. However, in PRESS2 Kenya, the window of time for approaching parents was within 6 h after death ensuring that MITS could be conducted within first 12 h, per the study protocol. In SIP Ethiopia, the study team realized that if there was a delay in approaching the family for MITS counselling, then a refusal of consent was likely because most families reside in rural areas and were in a hurry to reach the bus station with the body of the deceased child, to return home. In PURPOSe Pakistan, sometimes counsellors had to delay approaching the family for consent until the father arrived, as in most cases, the father was the decision maker for consenting for MITS.

- *Who obtains consent?*

In SIP Ethiopia, the consent was obtained by nurses with bachelor’s degrees and pediatric residents, both of whom were already actively involved in the patient management. The first person to approach the parents were the nurses, but if the parent seemed to be unwilling, the pediatric resident was engaged to facilitate the consent process. This approach was utilized as residents are perceived to be more credible by parents. In PURPOSe Pakistan, PURPOSe India, CHAMPS Bangladesh and PRESS2 Kenya, the counsellors, principally recruited for the MITS study project, obtained the consent from the parents. In a few cases, counsellors at the PURPOSe Pakistan and CHAMPS Bangladesh sites involved the attending physician and CHAMPS study physicians, respectively in counselling sessions to help build trust with the family, as the counsellors were not hospital staff.

- *Who gives consent?*

In SIP Ethiopia, only parents gave consent for the MITS procedure. However, in PURPOSe Pakistan, PURPOSe India and CHAMPS Bangladesh, extended family was also involved in the consent process including in-laws, grandparents, etc. However, in CHAMPS Bangladesh in most cases either the father or mother ultimately gave the final consent. In PRESS2 Kenya, most consent decisions were made by the parents; however, grandparents were also involved in a few instances.

- *Time taken by family/parents to make a decision*

In all five settings, the time taken by family members to make a decision varied from a few minutes up to 24 h. At times, parents agreed immediately during the counselling session; however, at other times the decision was made the next day following consultation with the family members. In PURPOSe India, the family members took 30–45 min on average to decide whether to consent to MITS. In SIP Ethiopia, when a father was not present during the counselling session, mothers typically waited until the father’s return before making the decision. In PURPOSe Pakistan, when extended family was involved in the consent process, at times it was difficult for all to agree and to come to a decision.

### Challenges encountered during counseling and consenting process

Several challenges were encountered during counseling and consenting process. In all five settings, the emotional turmoil and dynamics of grief reduced the families’ capacity to comprehend and understand the procedure and contributed to delays in the consenting process. Further, deaths occurred at all hours and counsellors found that in order to reach families immediately they had to approach them during the very early morning or late at night when they were especially exhausted and were frequently not in the optimal frame of mind to grasp the importance of MITS with regards to their loved one. In PURPOSe Pakistan, rapport building with families and particularly, with the mother was challenging because the counsellors were not directly part of the health facility compared to other sites where the study staff were also attending physicians and NICU/ER nurses employed by the facility. Also, counselling was challenging when the mother’s stay was short as in cases of stillbirth and early deaths of preterm births. Non- availability of both parents and other family members prolonged the process of counselling and decision making. In PURPOSe Pakistan, PURPOSe India, PRESS2 Kenya and CHAMPS Bangladesh, where extended family was involved in decision making, at times it was difficult to bring all family members to a consensus. In addition, the PURPOSe Pakistan team conducted counselling on two different floors [ground floors (ER) and 6th floor (nursery)] which made staffing administratively difficult to manage. In SIP Ethiopia and PURPOSe India, counsellors felt that many parents were unable to understand the study purpose in general and also the MITS procedure. In families with lower levels of education, counsellors found it quite difficult for them to really understand the rationale for conducting MITS. Generally, making a decision about MITS was challenging when there was only one parent present. At times, the mother was admitted to the intensive care unit (ICU) and it was difficult for the father to decide on his own. In the case where the father was not present at the time of death, the mother was not able to make a decision about the procedure, perhaps due to cultural reasons. In some cases, the parents’ religion was a barrier to consent. In PRESS2 Kenya, PURPOSe India and PURPOSe Pakistan, a few families and parents belonging to the Muslim religion refused to give consent for the MITS procedure as they believed that it was against their cultural values and not permissible in their religion.

### Facilitators for MITS consent process

In all five settings, counsellors found that higher levels of education within a family increased the likelihood they would understand the importance of the MITS procedure and facilitated consent. Also, use of local languages for explaining the procedure and study purpose facilitated the consent and decision-making process. In SIP Ethiopia, PURPOSe India, PURPOSe Pakistan and PRESS2 Kenya, two factors facilitating MITS consent included a fast turnaround time of the procedure, and the MITS technique being less invasive than conventional autopsy. In some cases, MITS was conducted while the parents were completing the discharge process, and the body was handed over to the parents for burial rituals at the same time as the parents were ready to leave the facility. Additionally, the SIP Ethiopia team and PURPOSe Pakistan team received Quranic evidence on permissibility of MITS procedure from the local Muslim religious leader to facilitate the counselling process. And as mentioned earlier, CHAMPS Bangladesh received a Fatwa, signifying that MITS was acceptable in the Muslim faith. However, neither site systematically assessed if that really influenced the decision making process of MITS. In PURPOSe Pakistan, the team observed that the presence of an attending physician during the counselling session facilitated the consent and decision-making process. In CHAMPS Bangladesh, the most common reason for giving consent for the MITS procedure included parents’ desire to know about the cause of child death, especially in cases where parents had already experienced multiple stillbirths and neonatal deaths.

## Conclusion

Our study described the counseling and consent methods and approaches used in MITS projects across five countries in Africa and South Asia. Key informant interviews were conducted with researchers to describe the characteristics and backgrounds of counsellors, the counselling environment and timing of consent and perceived facilitators and barriers encountered during the consent process. Despite the geographical and cultural differences between sites, some commonalities were observed. Ensuring that counsellors had the skills and knowledge necessary to establish relationships with parents and families was consistent across all five sites. All selected counsellors had backgrounds in social science, psychology and counseling or clinical expertise in obstetrics/gynecology or pediatrics. All sites reported providing training for counsellors in rapport building and offering emotional support to families. Counsellors also consistently found that using the local language to describe the MITS procedure helped to build rapport and facilitated consent at all sites. While the physical locations identified for MITS counseling varied between sites, only a minority of sites had a dedicated and private room for meetings with families. However, counsellors at all sites aimed to optimize the resources available to maintain parents’ privacy and confidentiality. In most sites, counsellors adapted the consent process to accommodate for extended family to take part in the decision making. Typically, this meant delaying consent until all family members could be engaged in the process. Most sites noted that the inclusion of extended family increased the complexity of counseling and sometimes achieving consensus among extended family had the potential to be a barrier to consent. However, counsellors across sites found that highlighting the less invasive nature of MITS and ensuring MITS would be conducted quickly so that parents could proceed with burial facilitated consent. The expansion of MITS globally creates opportunities to further study these commonalities, identify those that are sustained across broader contexts, and has the potential to inform MITS counselling approaches and strategies for the future.

## Data Availability

The datasets used and/or analyzed during the current study are available from the corresponding author on reasonable request.

## Abbreviations

MITS: Minimally invasive tissue sampling
SDG: Sustainable Development Goals
RTI: Research Triangle Institute
SIP: Study of Illness in Preterms
PRESS: Pediatric Respiratory Etiology Surveillance Study
PURPOSe: Project to Understand and Research Preterms and Stillbirths
CHAMPS: Child Health and Mortality Prevention Surveillance
ER: Emergency Room
NICU: Neonate intensive care unit
